# Characteristics of temporal patterns of cortisol and luteinizing hormone in primiparous, postpartum, anovular, suckled, beef cows exposed acutely to bulls

**DOI:** 10.1186/1477-7827-8-89

**Published:** 2010-07-20

**Authors:** Shaun A Tauck, Jesse R Olsen, Jarrod RC Wilkinson, Riley J Wedlake, Kathleen C Davis, James G Berardinelli

**Affiliations:** 1 Department of Animal and Range Sciences, Montana State University, 119 Linfield Hall, Bozeman, MT, 59717, USA

## Abstract

**Background:**

The physiological mechanism by which bulls stimulate resumption of ovarian cycling activity in postpartum, anovular, suckled cows after calving may involve the concurrent activation of the hypothalamic-hypophyseal-ovarian (HPO) axis and hypothalamic-hypophyseal-adrenal (HPA) axis. Thus, the objectives of this experiment were to determine if characteristics of temporal patterns of cortisol and luteinizing hormone (LH) in postpartum, anovular, beef cows are influenced by acute exposure to bulls. The null hypotheses were that daily, temporal characteristics of cortisol and LH concentration patterns do not differ between cows exposed acutely to bulls or steers.

**Methods:**

Sixteen cows were assigned randomly 67 +/- 4 (+/- SE) after calving to be exposed to bulls (EB, n = 8) or steers (ES, n = 8) 5 h daily for 9 d (D 0 to 8). Blood samples were collected daily from each cow via jugular catheters at 15-min intervals for 6 h from 1000 to 1600 h each day. The 5-h exposure period began 1 h after the start of the intensive bleeding period. Characteristics of cortisol and LH concentration patterns (mean, baseline, pulse frequency, pulse amplitude, and pulse duration) were identified by PULSAR analyses.

**Results:**

Mean cortisol concentrations decreased (P < 0.05) in cows in both treatments from D 0 to D 2. Thereafter, mean cortisol concentrations stabilized and did not differ (P > 0.10) between EB and ES cows. The decrease in mean cortisol concentrations in EB and ES cows from D 0 to D 2 was attributed to cows acclimatizing to intensive blood sampling and handling procedures. Consequently, analyses for characteristics of cortisol and LH concentration patterns included D 2 through 8 only. Cortisol mean and baseline concentrations, and pulse amplitude did not differ (P > 0.10) between EB and ES cows. However, cortisol pulse duration tended to be longer (P = 0.09) and pulse frequency was lower (P = 0.05) in EB than ES cows. LH pulse frequency was greater (P = 0.06) in EB than ES cows. All other characteristics of LH concentration patterns did not differ (P > 0.10) between EB and ES cows. Characteristics of cortisol concentration patterns were not related to characteristics of LH concentration patterns for ES cows (P > 0.10). However, as cortisol pulse amplitude increased, LH pulse amplitude decreased (b1 = -0.04; P < 0.05) for EB cows.

**Conclusions:**

In conclusion, exposing primiparous, postpartum, anovular, suckled cows to bulls for 5-h daily over a 9-d period did not alter mean concentrations of cortisol or LH compared to mean concentrations of cortisol and LH in cows exposed to steers. However, exposing cows to bull in this manner altered characteristics of temporal patterns of both LH and cortisol by increasing LH pulse frequency and decreasing cortisol pulse frequency. Interestingly, in cows exposed to bulls, as amplitude and frequency of cortisol pulses decreased, amplitudes of LH pulses increased and frequency of LH pulses tended to increase. Thus, the physiological mechanism of the biostimulatory effect of bulls may initially involve modification of the HPA axis and these changes may facilitate activation of the HPO axis and resumption of ovulatory cycles in postpartum, anovular, suckled cows.

## Background

Resumption of luteal function in primiparous, anovular, suckled beef cows after calving is accelerated if cows are exposed to bulls [1,2] or excretory products of bulls [3]. The mechanism for this effect appears to involve changes in the hypothalamic-hypophyseal-ovarian (HPO) axis to increase pulse frequency of luteinizing hormone (LH) concentrations in response to acute bull exposure (one 8-h exposure period) [4] or chronic bull exposure (24-h daily) [5]. Exposing cows to the excretory products of bulls stimulates resumption of ovarian cycling activity indicating that the mechanism for the biostimulatory effect of bulls is mediated by pheromones [3]. However, the effects of pheromones produced by bulls on neuroendocrine-endocrine events that precede this change in pulsatility of LH concentrations and stimulate resumption of ovarian cycling activity in postpartum, anovular, suckled cows are not well understood.

In rodents, male pheromones appear to influence female reproduction via the hypothalamic-hypophyseal-adrenal (HPA) axis [6]. Recent evidence from our laboratory has indicated that postpartum, anovular, suckled cows exposed to bulls resumed ovarian cycling activity sooner and had greater mean concentrations of cortisol than cows not exposed to bulls [7]. One interpretation of these results is that the HPA axis may be involved with the physiological pathway by which bull exposure stimulates resumption of ovarian ovulatory cycles in postpartum, anovular cows.

The objectives of this experiment were to determine whether acute exposure of postpartum, anestrous, suckled beef cows to bulls for 5-h daily over a 9-d period alters characteristics of temporal patterns of cortisol and LH concentrations. The null hypotheses tested were that mean concentrations of cortisol and LH and characteristics of temporal patterns of these hormones do not differ between postpartum, anovular, suckled cows exposed to bulls or to steers for 5-h daily over a 9-d period.

## Methods

This experiment was conducted at Montana State University Bozeman Area Research and Teaching Facility. Animal care, handling, and protocols used in this experiment were approved by the Montana State University Institutional Agricultural Animal Care and Use Committee.

### Animals and treatments

Sixteen 2-yr-old Angus × Hereford cows, two 4-yr-old Angus × Hereford bulls and two 1-yr-old Angus × Hereford steers were used in this experiment. Cows and calves were maintained in a single pasture for the calving season. Average calving date for these cows was February 4. Cows and calves had no contact with bulls or their excretory products for approximately 10 months before the start of this experiment. Interval from calving to the start of the exposure period was 67 ± 3.5 d (mean ± SE). Ovarian ovulatory activity before the start of the experiment was determined for each cow by ultrasound examinations of each ovary for the presence or absence of a corpus luteum at 12 and 2 d before the start of the experiment. Cows were visually monitored for estrous behavior daily from 0700 to 1830 throughout the experiment.

Two d before the start of the experiment cows were stratified by body weight, BCS, calf birth weight, calving date, sex of calf, and dystocia score, and assigned randomly to be exposed to either the two mature bulls (EB, n = 8) or the two steers (ES, n = 8). No cows with a dystocia score above 3 on a scale of 1 to 5 were used in this experiment (1 = no assistance, 5 = Caesarean section). Cows were exposed to bulls or steers for 5 h daily over a 9-d period (D 0 to 8).

### Facilities

**Figure 1:**
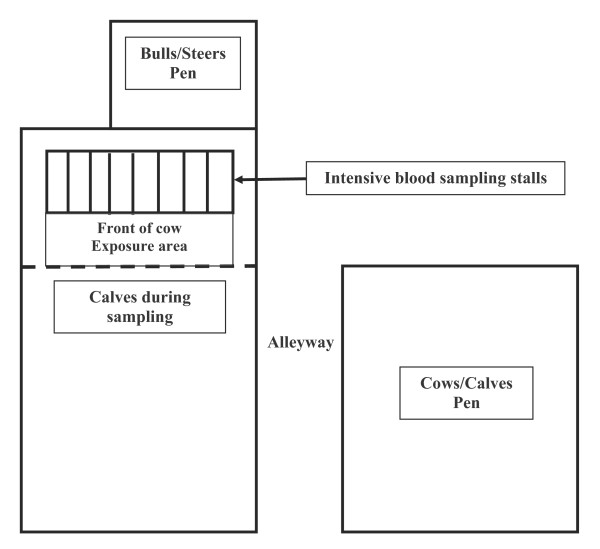
**Diagrammatic representation of the experimental layout for exposing cows to bulls or steers and for intensive blood sampling during the course of the experiment**. Two similar layouts were located 0.35 km from each other in a north to south orientation, The north layout was use for exposing cows to bulls and the south layout used for exposing cows to steers.

Cows were housed within pens in two separate lot areas separated by a distance of 0.35 km. Pens within the north lot were used to maintain EB cows while pens within the south lot were used to maintain ES cows. Pens dimensions were 41 m × 18 m (L × W). During each daily sample collection and exposure period, cows were moved into individual stalls within open-air sheds adjacent to each treatment groups' assigned pen. A diagrammatic representation of the layout of the stalls and pens for housing cows, calves, bulls, and steers is presented in Figure 1. Sheds were similar in structure, area and light density. Light density within sheds was monitored with a Minolta Autometer Pro Photometer at the level of heads of cows. Opaque tarps were used to manipulate light density so that cows in each sampling area were exposed to the same amount of light during each sampling period. During the daily sampling and exposure periods, cows were halter-restrained within side-by-side stalls (Figure 1). Bulls or steers were contained in the immediate vicinity in front of the cows; they were unrestrained and allowed to eat, roam, and come into contact with the frontal aspect of each cow. Calves were separated from cows immediately before cows were placed into stalls. Calves were housed in a pen approximately 15 m in front of cows and restrained from entering the stall area by a steel fence. Excretory products of bulls and steers were not cleaned from the exposure area during the experiment.

### Nutrition

Cows had free access to good quality, chopped mixed-grass alfalfa hay, and any pasture grasses that were available before the start of the experiment. Once cows and calves were moved into pens they were given free access to the same hay, 0.5 kg●hd^-1^● d^-1 ^cracked barley, water, and a trace mineral-salt supplement. The TDN of the diet exceeded the NRC requirement for lactating beef cows with a mature weight of 545 kg by approximately 15% [8]. Bulls were fed 0.5 kg●hd^-1^● d^-1^of cracked barley and good quality, chopped mixed-grass alfalfa hay. Steers were fed a finishing ration that consisted of 70% concentrate and 30% roughage throughout the experiment.

### Intensive blood sampling protocols

Two d before the start of the experiment each cow received an indwelling jugular catheter. Blood samples were collected daily from each cow in each treatment at 15-min intervals for 6 h (1000 to 1600 h) beginning 1 h before the 5-h exposure period each day during the 9-d period (D 0 to 8). The maximum number of 6-h sampling periods that could be collected each day was 16 (1 sampling period for each cow in the experiment) and the total number of sampling periods over the 9-d period was 72 for each treatment. At the beginning of each collection day catheters were cleared of heparinized saline before blood was collected and flushed after collection of each blood sample using sterilized physiological saline solution (0.9%). After collection of a sample, the catheter was again flushed with saline and capped. This procedure was repeated for each daily collection. Each catheter was flushed with heparinized-saline after collecting the last sample each day. Samples were refrigerated at 4°C and centrifuged at 1285 × *g *for 30 min at 4°C the following day. Sera was harvested and stored at -20°C until assayed for cortisol and LH. Blood samples were collected from cows in each treatment by the same technician throughout the experiment to reduce the possibility of variations in cortisol concentrations that may be associated with altering the human element of the environmental conditions.

### Resumption of ovarian cycling activity

Resumption ovarian cycling activity was monitored by assay of daily progesterone concentrations in samples collected 1 h after start of the 6-h sampling period. Serum was assayed for progesterone concentration in duplicate using solid-phase RIA kits (Siemens Medical Healthcare Diagnostics, Inc., Los Angeles, CA, USA) validated for bovine serum in our laboratory [1]. Intra-assay CV was less than 10% for serum pools. Progesterone concentration patterns were used to determine the occurrence of resumption of ovarian cycling activity and the intervals from the start of male exposure to resumption of ovarian cycling activity. An increase of progesterone concentration, above the average progesterone baseline of individual cows in three consecutive samples that exceeded 1 ng/mL was used to determine the occurrence of resumption of ovarian cycling activity. Intervals from the start of treatment to resumption of ovarian cycling activity were determined by the number of days from the treatment to the lowest inflection point before a rise in three consecutive samples that exceeded 1 ng/mL.

### Cortisol and luteinizing hormone (LH) assays

Cortisol concentrations in serum samples were assayed in duplicate using solid-phase RIA kits (Siemens Healthcare Diagnostics, Inc., Los Angeles, CA, USA) validated for bovine serum in our laboratory [7]. Intra- and interassay CV were < 10% for pools of postpartum cow sera that contained 135 and 25 ng/mL, respectively, of cortisol. Concentrations of LH in serum samples were assayed in duplicate by liquid-phase double antibody RIA [9]. The primary antibody was NIDDK anti-oLH-1 AFP 192279 Rb and bLH AFP11743 B was used for iodination and standards. Both assay reagents were obtained from the National Hormone and Pituitary Program (NHPP) and Dr. A. Parlow (University of San Francisco, San Francisco, CA). Intra- and interassay CV for sera pools that contained 12.5 ng/mL were 10.9% and 15.4%, respectively, and for sera pools that contained 1 ng/mL were 7.4% and 17.7%, respectively.

### Characteristics of temporal patterns of cortisol and LH concentrations

Characteristics of temporal patterns of cortisol and LH concentrations were determined by the Pulsar algorithm [10] using PC PULSAR (PC-Pulsar, Gitzen and Ramirez, University of Illinois). This algorithm identified characteristics of daily temporal patterns and included mean of all samples in a sampling period (mean concentration), mean of samples that were identified as baseline concentrations (baseline concentration), frequency (pulse frequency), mean for apex sample concentrations over all pulses in a sampling period (pulse amplitude), and mean number of minutes that sample concentrations within pulses were above baseline concentrations for a sampling period (pulse duration).

### Statistical analyses

Characteristics of temporal patterns of cortisol and LH concentrations were analyzed using PROC MIXED procedure for repeated measures of SAS (SAS Inst. Inc., Cary, NC). The model included treatment, day and the interaction of treatment and day. Animal was the experimental subject and day was the repeated measure. Means were separated by Bonferroni's multiple comparison tests.

Relationships between characteristics of temporal patterns of cortisol and LH concentrations within treatments were determined by regressing characteristics LH concentrations of all cows within a treatment without regard to day on respective characteristics of cortisol concentrations of all cows within a treatment without regard to day using the PROC REG procedure of SAS. So that mean concentrations of LH were regressed on mean concentrations of cortisol and baseline concentrations of LH were regressed on baseline concentrations of cortisol, and so on. The model was a standard regression model in which characteristics of temporal patterns of cortisol concentrations were used as the explanatory variable and characteristics of temporal patterns of LH concentrations were used as dependent variables.

## Results

None of the cows exposed to bulls (EB) or exposed to steers (ES) for 5-h daily during the 9 d of sampling resumed ovarian cycling activity during the experiment (0 of 8 (0%) and 0 of 8 (0%), respectively).

### Adaptation to handling and intensive blood sampling protocols

**Figure 2:**
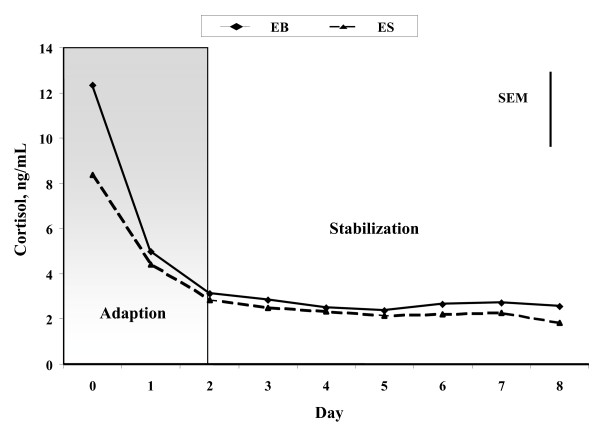
**Least squares means for daily mean concentrations of temporal patterns of cortisol in postpartum, anovular, suckled cows exposed to bulls (EB; n = 8) or steers (ES; n = 8) for 5 h daily over a 9-d intensive blood sampling period (D 0 = start of bull or steer exposure)**. The vertical bar represents the pooled standard error of the mean (SEM). The grey shaded box represents the period during which cows adapted to intensive blood sampling and handling protocols (D 0 to D 2). The period of stabilization represents D 2 to 8 after exposure.

There was no treatment or treatment by day interaction (P > 0.10) for mean cortisol concentrations of cows from D 0 to D 2. Mean concentrations of cortisol decreased (P < 0.05) from 10.2 +/- 1.7 ng/mL (+/- SE) on D 0 to 2.8 +/- 0.24 ng/mL on D 2 in both EB and ES cows (Figure 2). Thereafter, from D 2 through 8, daily mean concentrations of cortisol did not differ (P > 0.10) among days or between treatments (Figure 2); mean concentration of cortisol for all cows from D 2 through 8 was 2.5 +/- 0.10 ng/mL. The decrease in mean cortisol concentrations in EB and ES cows from D 0 to D 2 was attributed to cows adapting to stressors involved with handling and intensive blood sampling protocols used in this experiment. Consequently, analyses of treatment effects for characteristics of temporal patterns of cortisol and LH concentrations and regression analyses focused on data derived from temporal patterns of cortisol and LH concentrations from D 2 through 8 of the experiment.

### Characteristics of temporal patterns of cortisol concentrations, D 2 through D 8

There was no (P > 0.10) day effect or treatment by day interaction for characteristics of temporal patterns of cortisol concentrations from D 2 to D 8 of the experiment.

Baseline concentrations of cortisol and amplitudes of cortisol pulses did not differ (P > 0.10) between EB and ES cows from D 2 through 8 (Table 1). However, cortisol pulse frequency (pulses/h) was lower (P = 0.05) and pulse duration tended (P = 0.09) to be longer in EB cows than in ES cows (Table 1).

**Table 1:** Characteristics of temporal patterns of cortisol

	Treatment			
Variable	EB	ES	**SEM**^**a**^	*P *value
n	8	8		
Mean, ng/mL	2.9	2.5	0.9	> 0.10
Baseline, ng/mL	1.2	0.9	0.8	> 0.10
Pulse frequency/h	0.4	0.7	0.1	0.05
Pulse amplitude, ng/mL	6.1	5.6	1.2	> 0.10
Pulse duration, min	59.6	51.1	11.1	0.09

Least squares means of cortisol concentration patterns (mean, baseline, pulse frequency, pulse amplitude, and pulse duration) in blood samples collected at 15-min intervals between 1000 and 1600 h for postpartum, anovular, suckled cows exposed to bulls (EB) and cows exposed to steers (ES) from D 2 through D 8 of a 9-d exposure period. D 0 = Start of the experiment; cows were 67 +/- 3.5 d postpartum.

^a^SEM = Standard error of the mean.

### Characteristics of temporal patterns of LH concentrations, D 2 through D 8

There was no (P > 0.10) day or treatment by day interaction for characteristics of temporal patterns of LH concentrations from D 2 to D 8 of the experiment. Mean, baseline, pulse amplitude, and pulse duration of temporal patterns of LH concentrations did not differ (P > 0.10) between EB and ES cows from D 2 through 8 (Table 2). However, frequency of LH pulses was greater (P = 0.06) in EB cows than ES cows over D 2 through 8 of the exposure period (Table 2).

**Table 2:** Characteristics of temporal patterns of LH concentrations

	Treatment			
Variable	EB	ES	**SEM**^**a**^	*P *value
n	8	8		
Mean, ng/mL	0.7	0.6	0.2	> 0.10
Baseline, ng/mL	0.6	0.6	0.2	> 0.10
Pulse frequency/h	0.4	0.2	0.1	0.06
Pulse amplitude, ng/mL	0.9	0.6	0.5	> 0.10
Pulse duration, min	37.0	25.0	14.1	> 0.10

Least squares means of LH concentration patterns (mean, baseline, pulse frequency, pulse amplitude, and pulse duration) in blood samples collected at 15-min intervals between 1000 and 1600 h for postpartum, anovular, suckled cows exposed to bulls (EB) and cows exposed to steers (ES) from D 2 through D 8 of a 9-d exposure period.. D 0 = Start of the experiment; cows were 67 +/- 3.5 d postpartum.

^a^SEM = Standard error of the mean.

### Relationships between characteristics of temporal patterns of cortisol and LH concentrations

Linear regression of characteristics of temporal patterns of LH concentrations on characteristics of temporal patterns of cortisol concentrations indicated that characteristics of LH concentrations were independent of (P > 0.10) characteristics of cortisol concentrations within ES cows. However, regression analysis of pulse amplitudes of these hormones within EB cows indicated that as amplitudes of cortisol pulses increased, amplitudes of LH pulses decreased (b1 = -0.04 [ng/mL]/[ng/mL]; P < 0.05; Table 3). Furthermore, as frequency of cortisol pulses increased, frequency of LH pulses tended to decrease (b1 = -0.38 [pulses/h]/[pulses/h]; P = 0.06; Table 3), and as baseline concentrations of cortisol increased, baseline concentrations of LH tended to increase (b1 = 0.02 [ng/mL]/[ng/mL]; P = 0.09; Table 3). Mean concentrations and duration of cortisol pulses were not (P > 0.10) linearly related to mean concentrations or durations of LH pulses in EB cows (Table 3).

**Table 3:** Linear regression of LH characteristics on cortisol characteristics

Variable	Y-Intercept	**Slope**^**a**^	**R**^**2**^	*P *value
Mean, ng/mL	0.67	-0.01	0.01	0.10
Baseline, ng/mL	0.53	0.02	0.52	0.09
Pulse frequency/h	0.57	0.38	0.65	0.06
Pulse amplitude, ng/mL	1.19	-0.04	0.81	0.05
Pulse duration, min	36.5	-0.01	0.06	0.10

Linear regressions of characteristics of LH concentration patterns on characteristics of cortisol concentration patterns (mean, baseline, pulse frequency, pulse amplitude, and pulse duration) in blood samples collected at 15-min intervals between 1000 and 1600 h for postpartum, anovular, suckled cows exposed to bulls (EB) from D 2 through D 8 of a 9-d exposure period. D 0 = Start of the experiment; cows were 67 ± 3.5 d postpartum.

^a^Units for slopes are change in characteristic of LH per change in characteristic of cortisol.

## Discussion

The focus of this experiment was to evaluate the adrenal response and HPO axis activity in primiparous, postpartum, anestrous, suckled cows during acute exposure to bulls. In the present experiment, mean cortisol concentrations of cows were 10.2 ng/mL on D 0 and decreased to 2.5 ng/mL from D 2 through D 8. These values are close to and consistent with previous reports for systemic circulating cortisol concentrations in postpartum cows 40 to 60 d after calving, which ranged from 8 to 16 ng/mL [11-13]. Data for cortisol concentrations in the present study indicated that primiparous, postpartum, anestrous, suckled cows adapted to handling and sampling procedures within 2 d after the start of the experiment. Furthermore, characteristics of temporal patterns of cortisol concentrations, such as mean and baseline concentrations and amplitudes of pulses, did not differ between cows exposed to bulls or steers during the adaptation period. These results indicated that cows were not subjected to any undue stress throughout the experiment and that environmental conditions were similar for cows exposed to bulls and steers. However, mean concentrations of cortisol during the "adaptation" period (D 0 and D 1) were significantly greater and more variable in cows of both treatments than during the "stabilization" period (D 2 through 8). Echternkamp [14] reported that acute stress decreased mean concentrations of LH and frequency of LH pulses if cows showed a ten- to twenty-fold increase in cortisol concentrations. Thus, higher and more variable concentrations of cortisol during the "adaptation" period may have masked or artificially inflated potential differences between treatments. Therefore, data for cortisol and LH concentrations during the adaptation period were excluded from the analyses of characteristics of temporal patterns of hormone concentrations to better assess the effect of acute bull exposure.

Mean concentrations of cortisol in postpartum, anestrous cows did not differ between cows exposed to bulls and steers 5 h daily for 9 d. This result is inconsistent with those of Tauck et al. [7], in which anovular cows exposed to bulls for 30 d starting 58 d after calving resumed ovarian cycling activity sooner and had greater mean cortisol concentrations 9 d after the start of bull exposure than cows not exposed to bulls. However, the increase in cortisol concentrations for cows responding to the biostimulatory effect of bulls observed by Tauck et al. [7] could have been due to an increase in cortisol concentrations associated with the expression of estrus. Humphrey et al. [15] reported that cortisol concentrations increased from less than10 ng/mL during a 10-d period before estrus to greater than 40 ng/mL during a 3-d period after estrus in postpartum cows during the transition from anestral to estrual states. Therefore, the increase in mean cortisol concentrations observed in cows responding to the biostimulatory effect of bulls in the experiment by Tauck et al. [7] could have been influenced by ovulation, formation of corpus hemorrhagicum, and expression of estrus and not associated with the presentation of bull-pheromonal stimuli to cows. In light of this observation and the results of the present experiment, it appears that pheromonal stimuli presented by bulls may not induce an increase in mean concentrations of cortisol in postpartum, anestrous cows exposed to bulls.

The most significant results of this study were that duration of cortisol pulses tended to be longer and frequency of pulses were reduced in cows exposed to bulls than in cows exposed to steers 5 h daily for 9 d. One interpretation of these results is that bulls produce pheromones that immediately alter adrenal function and/or regulation over the short-term. This interpretation is much like the effect of male pheromones on physiological responses in females and is consistent with data from Mora and Sanchez-Criado [6] who reported that male urine sprayed into the nasal cavity of ovariectomized female rats stimulates ACTH release from the pituitary, which causes adrenal release of progesterone and corticosterone. Thus, pheromones produced by bulls and presented to cows acutely (5 h daily) appear to alter adrenal function and/or regulation of pituitary function in primiparous, postpartum, anestrous, suckled cows.

The increase in duration and decrease in frequency of cortisol pulses observed in postpartum, anestrous cows exposed to bulls may not have been due exclusively to cows sensing pheromones and responding to the biostimulatory effect of bulls, but may have been influenced by cows transitioning from anestrus to ovarian cycling activity. Berardinelli and Joshi [16] reported that the mean interval to resumption of ovarian cycling activity for cows exposed to bulls 55 d after calving was 16 d. Based on this result it is logical to assume that some cows in the present experiment that were exposed to bulls at 67 d after calving started to transition from anestrus to ovarian cycling activity during the 9-d experimental period.

The second objective of this experiment was to evaluate characteristics of temporal patterns of LH concentrations in cows exposed to bulls or steers 5 h daily for 9 d. The results indicated that LH mean and baseline concentrations, pulse amplitude, and pulse duration were not affected in cows exposed to bulls. However, cows exposed to bulls had greater LH pulse frequency than cows exposed to steers under the conditions of this experiment. This observation is consistent with Fernandez et al. [5] who reported that LH pulse frequency was greater in cows exposed to bulls for 2 h every 3 d for 18 d than in cows not exposed to bulls. Furthermore, Roelofs et al. [4] reported that LH pulse frequency was greater in dairy cows exposed to bulls for a single 8-h exposure period than during an 8-h exposure period on the previous day. Thus, it appears that bull exposure has an immediate effect on the hypothalamic-pituitary-ovarian (HPO) axis to cause increased LH pulse frequency in primiparous, postpartum, anestrous, suckled cows.

The final and perhaps most important objective of this experiment was to determine if characteristics of temporal patterns of cortisol concentrations are related to characteristics of temporal patterns of LH concentrations. The results indicated that there was no relationship between characteristics of temporal patterns of cortisol and LH concentrations for cows exposed to steers over the 9-d experimental period. However, in cows exposed to bulls, as amplitude and frequency of cortisol pulses decreased, amplitudes of LH pulses increased and frequency of LH pulses tended to increase. These results are consistent with McFarlane et al. [17] and Breen and Karsch [18] who reported that frequency and amplitude of LH pulses were decreased by infusing cortisol into intact and ovariectomized ewes, and indicate that cortisol has an inhibitory effect on LH secretion. In light of these results and results of the present experiment that frequency of cortisol pulses was lower and duration of cortisol pulses tended to be longer in cows exposed to bulls suggest that changes in the HPA axis caused by the biostimulatory effect of bulls may facilitate changes in the HPO axis and accelerate resumption of ovarian cycling activity in primiparous, postpartum, anestrous, suckled, beef cows.

## Conclusions

In conclusion, bull exposure for 5 h daily over a 9-d period did not appear to alter mean concentrations of cortisol or LH; however, bull exposure altered temporal patterns of cortisol and LH concentrations by decreasing frequency of cortisol pulses, tending to increase duration of cortisol pulses, and increasing frequency of LH pulses. Even though no biostimulatory effect, i.e., resumption of ovarian cycling activity, was observed in cows within the 9-d experiment, these results indicate that the presence of bulls for 5 h daily over a 9-d period altered the activity of the HPO axis and influenced adrenal function and/or regulation. Therefore, bulls may release a pheromone(s) that has an immediate effect on activity of the HPO axis and adrenal function and/or regulation of primiparous, postpartum, anestrous, suckled cows. This effect may facilitate or support the physiological mechanism whereby the biostimulatory effect of bulls accelerates resumption of ovarian cycling activity in primiparous, postpartum, anestrous, suckled cows.

## Competing interests

The authors declare that they have no competing interests.

## Authors' contributions

SAT, JRO, JRCW, and JGB took part in the design the experiment, performed the experiment and conducted assays, analyzed data, and drafted this manuscript. RJW assisted in handling of animals and their care during sample collection, sample collection and processing. KCD contributed to the analyses of experimental data. All authors read and approved the final manuscript.
